# Canadian food ladders for dietary advancement in children with IgE-mediated allergy to milk and/or egg

**DOI:** 10.1186/s13223-021-00583-w

**Published:** 2021-08-05

**Authors:** Alanna Chomyn, Edmond S. Chan, Joanne Yeung, Timothy K. Vander Leek, Brock A. Williams, Lianne Soller, Elissa M. Abrams, Raymond Mak, Tiffany Wong

**Affiliations:** 1 Department of Pediatrics, Division of Clinical Immunology & Allergy, University of British Columbia, BC Children’s Hospital, Room 1C31B, 4480 Oak Street, Vancouver, BC V5Z 4H4 Canada; 2 Pediatric Allergy & Asthma, Department of Pediatrics, University of Alberta, Edmonton, AB Canada; 3 Food, Nutrition, and Health, Faculty of Land and Food Systems, University of British Columbia, Vancouver, BC Canada; 4 Department of Pediatrics and Child Health, Section of Allergy and Immunology, University of Manitoba, Winnipeg, Canada

**Keywords:** Food ladders, Food allergy, Cow’s milk allergy, Egg allergy, Oral immunotherapy

## Abstract

Food ladders are clinical tools already widely used in Europe for food reintroduction in milk- and egg-allergic children. Previously developed milk and egg ladders have limited applicability to Canadian children due to dietary differences and product availability. Herein we propose a Canadian version of cow’s milk and egg food ladders and discuss the potential role that food ladders may have in the care of children with IgE-mediated allergies to cow’s milk and/or egg, as either a method of accelerating the acquisition of tolerance in those who would outgrow on their own, or as a form of modified oral immunotherapy in those with otherwise persistent allergy.

## To the editor,

Cow’s milk and egg are among the most common food allergies in young children. IgE-mediated milk and egg allergies are not only significant causes of food-induced anaphylaxis in children but are associated with numerous other adverse medical and psychosocial outcomes including nutritional and growth concerns and impaired quality of life for both children and their caregivers [1–4]. Although milk and egg allergies have historically been regarded to have a good prognosis, with many children outgrowing their food allergy in childhood, recent studies suggest that the rate of resolution may be slowing over time, with only 50% resolution by 5–6 years of age and increasing persistence of these allergies into adolescence or adulthood [5–7]. High baseline sIgE levels, such as those greater than 10kU_A_/L for egg and milk, may predict persistence of allergy [5–7]. Management of food allergies has historically been limited to avoidance with periodic reassessment. However, there is increasing recognition that children with egg and milk allergy may tolerate baked/processed forms of milk and egg, and that ongoing ingestion of these forms may help with resolution of their food allergy [8, 9]. Conformational changes in immune-activating epitopes that occur during the baking or heating processes alter the allergenicity of milk and egg and may allow for tolerance [10].

Milk and egg ladders (henceforth called food ladders) are tools designed to guide patients through a home-based gradual stepwise introduction of increasingly allergenic forms of milk and egg in a demedicalized setting. Originally designed in the United Kingdom for the management of non-IgE-mediated food allergies, food ladders extrapolate from previous evidence that the vast majority of egg- and milk-allergic children are able to tolerate extensively heated forms of these allergens, such as in baked goods [11–15]. Regular ingestion of tolerated forms of milk and egg may induce accelerated tolerance, allowing liberalization of the diet to more allergenic forms of the food over time. Food ladders are now widely used in Europe for this purpose and are included in the British Society for Allergy & Clinical Immunology’s guidelines for the management of egg allergy [16]. According to a 2017 survey of 114 healthcare professionals from around the globe, 68% of respondents reported that they utilized milk ladders [17]. In Canada, food ladders appear to be increasingly being adopted by allergists.

Despite increasing use, there is a paucity of published research on food ladders. Ball and Luyt studied the role of milk ladders in 86 milk-allergic children with a history of mild reactions to milk [18], and ultimately 91% of children in their study were able to tolerate the majority of dairy products within 4–6 months. While 43% experienced minor adverse reactions, there were no cases of anaphylaxis. To our knowledge, there are no studies published to date describing egg ladder use.

**Fig. 1:**
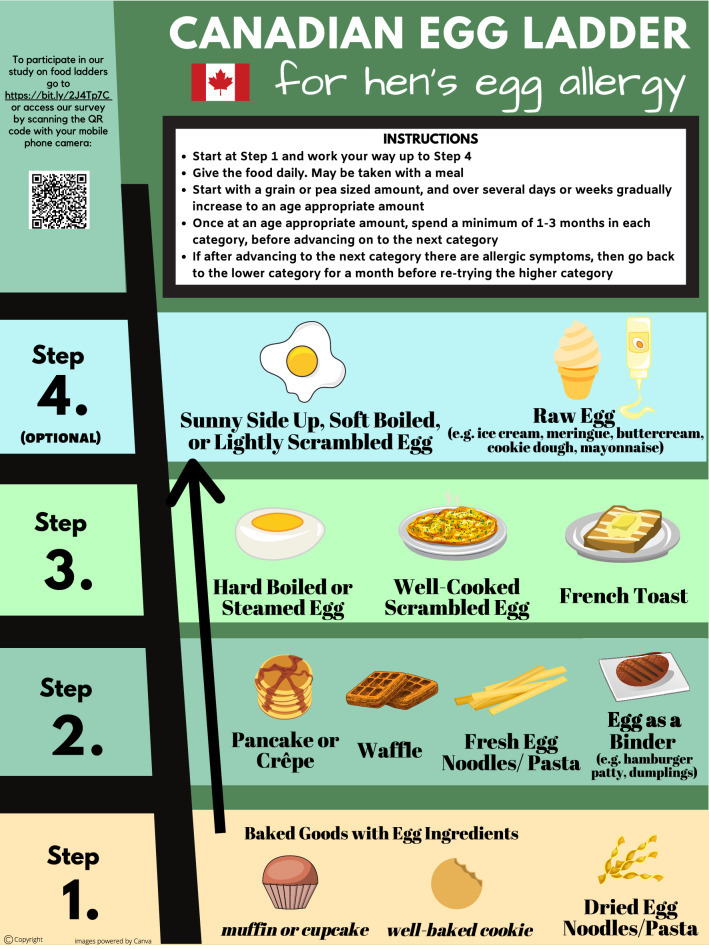
Canadian egg ladder

**Fig. 2:**
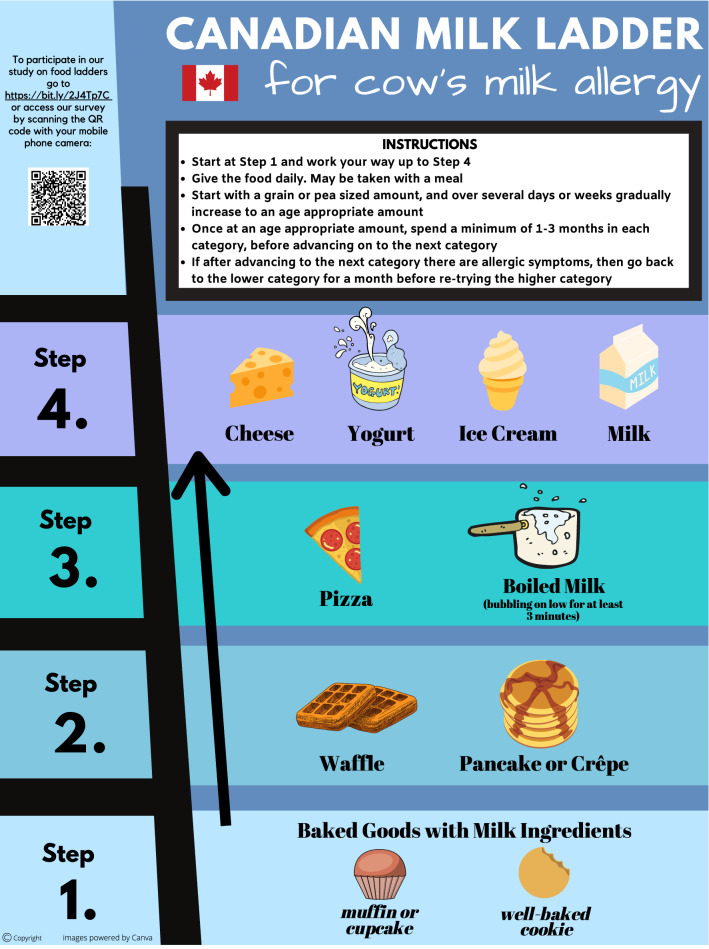
Canadian  milk ladder

European versions of food ladders have limited applicability to the Canadian diet, as they include foods that may be seldomly consumed in many Canadian households. Hence, we developed the Canadian Food Ladders using foods more typically consumed by Canadian children [19, 20] (Figs. 1, 2).

There are four “Steps” in each ladder, with the least allergenic forms of milk or egg in Step 1, progressing to the most allergenic forms in Step 4. Children are typically introduced to their relevant allergen at Step 1, starting with a grain- or pea-sized amount of food. If tolerated, the child should consume the food on a daily basis. The serving size offered is gradually increased as tolerated over several days to weeks until an age-appropriate amount is reached. We advise that children continue to consume age-appropriate serving sizes of foods at that step on a daily basis for at least 1–3 months before advancing to the next step in the ladder. If IgE-mediated allergy symptoms occur with the introduction of a new food on the ladder, the child should return to consuming previously tolerated foods for at least 1 month before again cautiously attempting to advance on the ladder. Parents should be counselled by the allergist overseeing their child’s food ladder use on how to recognize and manage allergic reactions. If a child is confirmed to be fully tolerant to foods on a higher step of the ladder, they need not start at Step 1; rather, they may start at the step corresponding to foods currently tolerated. Caregivers are advised that children can progress as slowly through the food ladder as tolerated and desired, as even consuming baked goods regularly (Step 1) has been shown to promote tolerance [8, 9].

The Canadian Food Ladders are intended for use in preschool-aged children with a history of only mild IgE-mediated reactions to milk and/or egg. The use of food ladders is likely safest in preschool-aged children based on safety data extrapolated from studies on oral immunotherapy revealing higher rates of anaphylaxis in older children compared to preschoolers [21, 22]. Contraindications to the use of food ladders include a previous life-threatening episode of anaphylaxis or asthma that remains inadequately controlled on medium dose inhaled steroid therapy. Relative contraindications include both medical and socioeconomic factors, such as a recent severe asthma exacerbation, a language barrier or cognitive impairment. Food ladders should be initiated at the recommendation of an allergist, and patients using a food ladder should receive regular follow up (we suggest at least every 6 months).

Data in regard to efficacy of oral immunotherapy for food allergies has been promising, with excellent safety and effectiveness in the preschool aged group [21, 23]. We propose that food ladders be considered a modified form of oral immunotherapy for preschoolers with very high baseline sIgE levels or older children, representing phenotypes that would be unlikely to outgrow their allergy via strict avoidance. Similar to oral immunotherapy, food ladders consist of the regular administration of small doses of food allergen and likely lead to similar immune changes that assist in establishing tolerance. In addition, food ladders have the added benefit of allowing children to gradually expand their diet, whether by promoting tolerance or following the natural progression of resolution of their food allergy in a home setting, while potentially using fewer healthcare resources than other models of oral immunotherapy delivery (especially due to the lack of need for oral food challenges or multiple visits for conventional oral immunotherapy with the unheated food). Similar to other models of oral immunotherapy, food ladders have the potential to alleviate food-allergy related anxiety and improve quality of life for families and their children with milk and egg allergy.

However, while food ladders are a promising tool for facilitating dietary expansion for children with milk or egg allergies, further research is needed to improve confidence with their use. Further safety and efficacy data are needed, particularly for the egg ladder where this data is mainly extrapolated from baked egg ingestion and oral immunotherapy studies. Additionally, with further study, this concept may ultimately prove safe and appropriate for older children and adults. And although we propose that food ladders be considered a modified form of oral immunotherapy, long term data is needed to establish whether their use truly increases reaction thresholds and protects against potential accidental exposures. Finally, qualitative data from patients and evaluation of the impact of food ladder use on quality of life and food allergy-related anxiety is also needed. As such, our group is currently examining the experience of Canadian allergists and their patient families with the Canadian Food Ladders.

## Conclusion

Food ladders offer a flexible and proactive approach to management of lower risk egg- or milk-allergic children. They have the potential to facilitate gradual dietary expansion and accelerate the resolution of allergy. For children with persistent allergy beyond the preschool age, we propose that food ladders be considered a modified form of oral immunotherapy. While food ladders are not appropriate for use in all children with egg and milk allergies, they are a promising tool with evidence supporting efficacy and safety extrapolated from studies on oral immunotherapy as well as from limited studies of milk ladder use.

## Data Availability

Not applicable.
